# Effect of probiotic *Lactobacillus* on lipid profile: A systematic review and meta-analysis of randomized, controlled trials

**DOI:** 10.1371/journal.pone.0178868

**Published:** 2017-06-08

**Authors:** Yucheng Wu, Qingqing Zhang, Yin Ren, Zhongbao Ruan

**Affiliations:** 1 Department of Cardiology, Taizhou People's Hospital, Taizhou, Jiangsu, China; 2 Department of Endocrinology, Taizhou People's Hospital, Taizhou, Jiangsu, China; Universita degli Studi di Milano, ITALY

## Abstract

**Objective:**

To assess the efficacy of probiotic *Lactobacillus* on serum lipids using a meta-analysis of randomized, controlled trials.

**Methods:**

Fifteen studies containing 15 trials, with 976 subjects were included. The pooled WMD was calculated by random effects model.

**Results:**

Probiotic *Lactobacillus* consumption significantly reduced TC by 0.26mmol/l (95% CI, -0.40 to -0.12) and LDL-C by 0.23mmol/l (95% CI, -0.36 to -0.10). Subgroup analysis of trials found significantly reduction of TC using *L*. *plantarum* and reduction of LDL-C using *L*. *plantarum* or *L*. *reuteri*. No significant effects were found on TG and HDL-C levels after supplementation with probiotic *Lactobacillus*. While, subgroup analysis found significantly beneficial effects on TG and HDL-C by consuming synbiotic food, containing *L*. *sporogenes* and inulin.

**Conclusion:**

Consuming probiotic *Lactobacillus*, especially *L*. *reuteri* and *L*. *plantarm*, could reduce TC and LDL-C significantly. The study also suggested significantly beneficial effects on TG and HDL-C by consuming synbiotic food, containing *L*. *sporogenes* and inulin.

## Introduction

As we know, coronary artery disease (CAD) is one of the most important causes of deaths all over the world. Epidemiological reports have demonstrated that the high risk of CAD is associated with dyslipidemia, especially the high level of low density lipoprotein-cholesterol (LDL-C)[1]. One of the considerable first line for treating dyslipidemia is dietary regulations and recommendations; however, using these methods, only a modest amelioration can be achieved[2].

Probiotics are called as live microorganisms which are beneficial to human health when consumed in adequate quantities [3]. Specific strains of probiotic bacteria, such as *Lactobacillus*, have been demonstrated to improve anti-diarrheal and anti-inflammatory, potentiate immune, and delay diabetes[4]. Several *Lactobacillus* strains have been found to reduce total cholesterol (TC) and triglyceride (TG) concentrations in rats[5, 6], while in human clinical studies there is no consensus on the effects of consumption of *Lactobacillus* strains on lipid profile. One previous study has shown that three weeks daily administration of 200ml milk containing *Lactobacillus acidophilus* L1 was associated with the reduction of TC level in hypercholesterolemic individuals[7]. Further, Fuentes *et al*. reported that daily intake of *L*. *plantarum*in a capsule containing 1.2×10^9^ CFU lowered TC and LDL-C concentrations in participants with hypercholesterolemia after 12 weeks[8]. However, Hove *et al*. reported that 12 weeks intake of milk fermented with *L*. *helveticus* had no effect on serum lipids in type 2 diabetes patients[9]. As we know, there has been no meta-analysis on the efficacy of probiotic *Lactobacillus* for lipid control. In this paper, a meta-analysis was carried out to assess the functional effects of supplementation of probiotic *Lactobacillus* on lipid profile, which may provide further information on the efficacy of specific strains of *Lactobacillus* required to lipid control.

## Experimental section

### Literature search

Studies exploring the effects of probiotic *Lactobacillus* consumption on serum lipid were searched in PubMed, Web of Knowledge, Cochrane Library, and Embase databases. The search was last updated in July 2016 and involved only full-text articles published in English. The search was performed, for example in PubMed, as following: (((((((((((((((cholesterol) OR (((("Cholesterol"[Mesh]) OR "Cholesterol, VLDL"[Mesh]) OR "Cholesterol, LDL"[Mesh]) OR "Cholesterol, HDL"[Mesh])) OR plasma lipids) OR triglycerides) OR "Triglycerides"[Mesh]) OR serum lipids) OR HDL-C) OR LDL-C) OR low density cholesterol) OR high density lipoprotein cholesterol) OR low density lipoprotein cholesterol) OR very low density lipoprotein cholesterol) OR VLDL-C)) AND (("*Lactobacillus*"[Mesh]) OR *Lactobacillus*)) AND (("Randomized Controlled Trial" [Publication Type]) OR "Randomized Controlled Trials as Topic"[Mesh]). All the references of included articles were also established by a hand search.

### Study eligibility

According to the PRISMA guidelines, the inclusion criteria was as following: (1) randomized controlled trial (RCTs), (2) adults, (3) the serum lipid changes and standard deviation (SD) were available, (4) interventions of the articles only used specific strains of *Lactobacillus*, (5) articles were published in English language. Exclusion criteria: (1) unpublished reports, (2) review articles, case reports, editorials, and letters, (3) not include a control group.

### Data extraction

**Fig 1:**
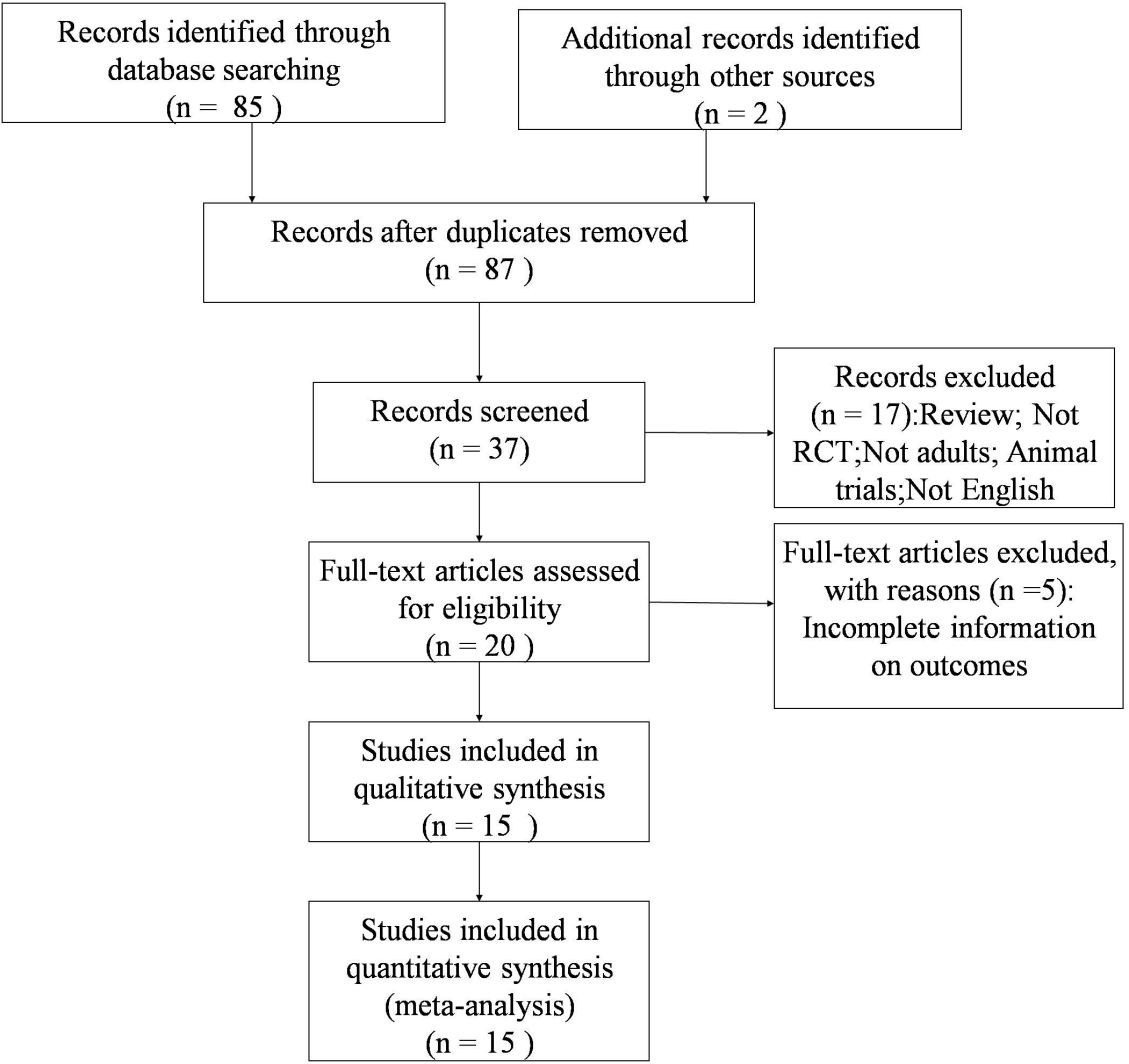
The study flow diagram.

Firstly, initial screening of studies was done independently by two researchers. Then, abstracts and full texts were reviewed according to the eligibility criteria. Finally, the included articles were determined by the two reviewers together. The data were extracted from each study as following: the first author, study design, publication year, characteristics of subjects, species of *Lactobacillus* strains, dose and duration of *Lactobacillus* strains supplementation, and baseline measures and changes from baseline of serum lipid. When the study has two or more comparisons, we chose the one whose follow-up period is the longest. There is no restriction on minimum number of interventions or controls in our meta-analysis. The study flow is showed as Fig 1. Wu YC and Zhang QQ conducted the search, data extraction, and analysis of study quality.

### Risk of bias

S1 Table shows the risk of bias of included articles, which was assessed by the Cochrane Collaboration’s tool. Two studies clearly describe the method of randomization (13.3%, n = 2). The risk of bias regarding concealed allocation and selective reporting was unclear (100%, n = 15). Low risk of bias was revealed on incomplete outcome data (93.3%, n = 14), blinding of participants (100%, n = 15), and other sources of bias (93.3%, n = 14).

### Statistical analysis

SD of the changes of serum lipid from baseline, if not reported, was calculated using the formula below (1). Statistical analysis was conducted using STATA 11.0 (Stata, College Station, TX, USA). The effect of *Lactobacillus* strains on serum lipid was showed as the weight mean difference (WMD) of serum lipid changes between the treatment groups and control groups, which was calculated as the difference in the mean outcome between the groups divided by SD of outcome among participants and analyzed by the random effects model regardless of heterogeneity. Heterogeneity across the included studies was assessed using the I^2^ statistics, representing the percentage of actual variation in relation to total variation. Subgroup analysis was also performed. P < 0.05 was considered statistically significant. Additionally we assessed the probability of publication bias with Begg’s funnel plots and the Egger’s test, with p value < 0.10 considered representative of statistically significant publication bias.

$${SD}_{change}=\sqrt{{{SD}_{baseline}}^{2}+{{SD}_{final}}^{2}-(2\times 0.8\times {SD}_{baseline}\times {SD}_{final})}$$ (1)

## Results

### Characteristics of included studies

Fig 1 shows the search flow for this meta-analysis. The literature search yielded 87 citations, of which 37 articles were retrieved after preliminary screening. Finally, 15 articles were included in this meta-analysis[7–21].

Fifteen studies, with 976 subjects in all, were included in this meta-analysis. All the included studies were randomized, controlled trials with double-blind design. Of the 15 studies, 13 were parallel and the other 2 were cross-over.

Table 1 shows the basic characteristics of included trials. Of the 15 studies, 5 used subjects with hypercholesterolemia, 3 were healthy participants, 2 were diabetes mellitus patients, 1 included smokers, 2 included obese subjects, 1 included pregnant women and 1 included patients with gestational diabetes mellitus. Of the 15 studies, 13 studies used probiotics, including *L*. *plantarum*, *L*. *acidophilus*, *L*. *fermentum*, *L*. *helveticus*, *L*. *salivarius*, *L*. *reuteri*, and *L*. *rhamnosus*, and the other 2 studies used synbiotics containing *L*. *sporogenes* and inulin. The duration and dose of probiotic *Lactobacillus* consumption varied between trials. The total daily dose changed from 10^7^ to 10^11^ colony-forming units. The duration changed from 3 to 24 weeks. To be mentioned, different trials give the different definitions about hypercholesterolemia that we didn’t make a subgroup analysis, so it is not quite necessary to give a new definition.

**Table 1:** Characteristics of included trials.

Study (publish year)	Design	Participants	Intervention	duration (wk)
Bukowska, H.(1998)	DB, PC, P	30 HC (42.6±2.8 y)	95:5 (v/v) fruit and fermented oatmeal soups and contains: 5×10^7 CFU/ml of Lactobacillus plantarum 299 v, 80 mg/dl oat fibers and rose hip drink	6
Schaafsma, G.(1998)	DB, C	30 healthy (33–64 y)	two strains of Lactobacillus acidophilus and contained 2.5% fructo-oligosaccharides, 0.5% vegetable oil and 0.5% milk fat. (10^7–10^8 CFU/g)	3
Anderson, J.W.(1999)	DB, C	40 HC (52–61 y)	Fermented Milk containing the L. acidophilus L1 strain (L1 FM)	4
de Roos, N.M.(1999)	DB, PC, P	78 healthy (18–65 y)	Lactobacillus acidophilus L-1(4.8×10^9 to 2.7×10^10 CFU)	6
Naruszewicz, M.(2002)	DB, PC, P	36 smokers (35–45 y)	Lactobacillus plantarum 299v (2×10^9 CFU)	6
Simons, L.A.(2006)	DB, PC, P	44 HC (30–75 y)	Lactobacillus fermentum(4×10^9 CFU)	8–10
Jones, M.L.(a)(2012)	DB, PC, P	127 HC (20–75 y)	Lactobacillus reuteri NCIMB 30242, 2.0× 10^9 CFU	9
Jones, M.L.(b)(2012)	DB, PC, P	131 healthy (20–75 y)	L. reuteri NCIMB 30242, 2.9×10^9 CFU	9
Fuentes, M.C.(2013)	DB, PC, P	60 HC (18–65 y)	Lactobacillus plantarum CECT 7527, 7528 and 7529, 1.2 ×10^9 CFU	12
Sharafedtinov, K.K.(2013)	DB, PC, P	40 obesity (30–69 y)	L. plantarum TENSIA, 1.5x10^11 CFU/gx50g	3
Taghizadeh, M.(2014)	DB, PC, P	52 pregnant women (18–35 y)	Lactobacillus sporogenes, 1×10^7 CFU, 0.04 g inulin (HPX)/g as the prebiotic	9
Shakeri, H.(2014)	DB, PC, P	52 DM (35–70 y)	L. sporogenes, 1.2×10^10 CFU	8
Hove, K.D.(2015)	DB, PC, P	41 DM (40–70 y)	300 ml L. helveticus Cardi04 yogurt	12
Lindsay, K.L.(2015)	DB, PC, P	100 women with gestational DM, >18 y	Lactobacillus salivarius UCC118	4–6
Sanchez, M.(2014)	DB, PC, P	125 obesity (18–55 y)	Lactobacillus rhamnosus CGMCC1.3724, 1.6 × 10^8 CFU with oligofructose and inulin	24

C, cross-over; CFU, colony-forming unit; DB, double blind; HC, hypercholesterolemia; P, parallel; PC, placebo control; and DM, diabetes mellitus.

### Main outcomes

**Fig 2:**
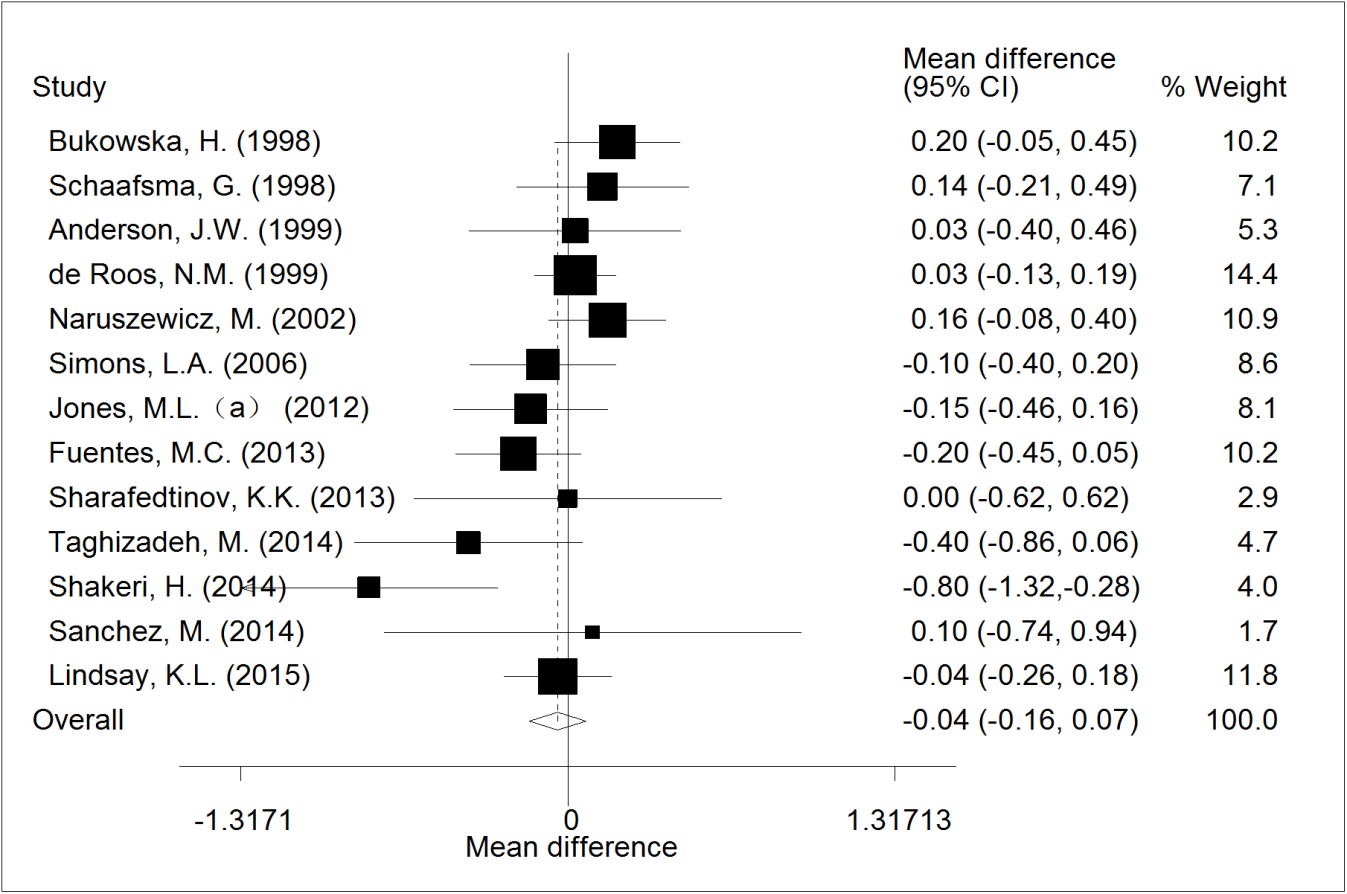
The effect of consumption of probiotic *Lactobacillus* on TG.

Of 13 RCTs, a random-effects meta-analysis did not show the effect of supplementation with probiotic *Lactobacillus* on TG levels (WMD = -0.04; CI,-0.16 to 0.07; p = 0.481). The forest plot of the effect was presented in Fig 2. The included studies showed highly significant homogeneity (I^2^ = 42.0%; p = 0.055).

**Fig 3:**
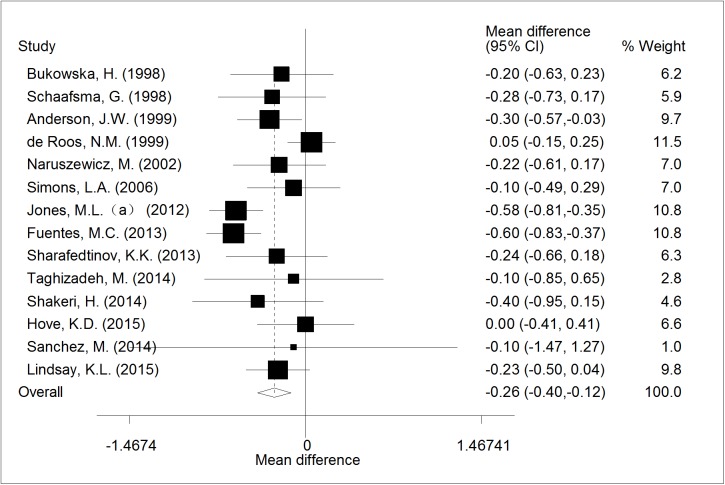
The effect of consumption of probiotic *Lactobacillus* on TC.

Of 14 RCTs, the meta-analysis result showed a significant reduction of 0.26mmol/l (95% CI, -0.40 to -0.12; p < 0.001) in TC compared with control (Fig 3), the heterogeneity of the included studies was moderate (I^2^ = 53.6%; p = 0.033).

**Fig 4:**
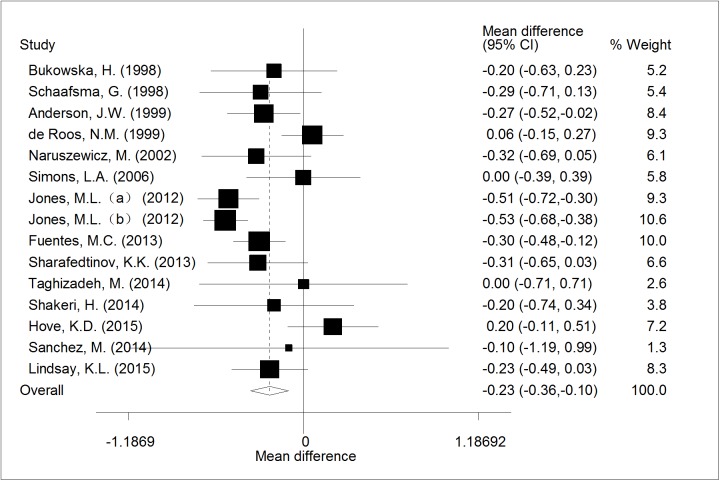
The effect of consumption of probiotic *Lactobacillus* on LDL.

A significant reduction of LDL-C by 0.23mmol/l (95% CI, -0.36 to -0.10; p< 0.001)was showed after meta-analysis of 15 trials, with high heterogeneity (I^2^ = 62.8%; p = 0.034). The forest plot of the effect was presented in Fig 4.

**Fig 5:**
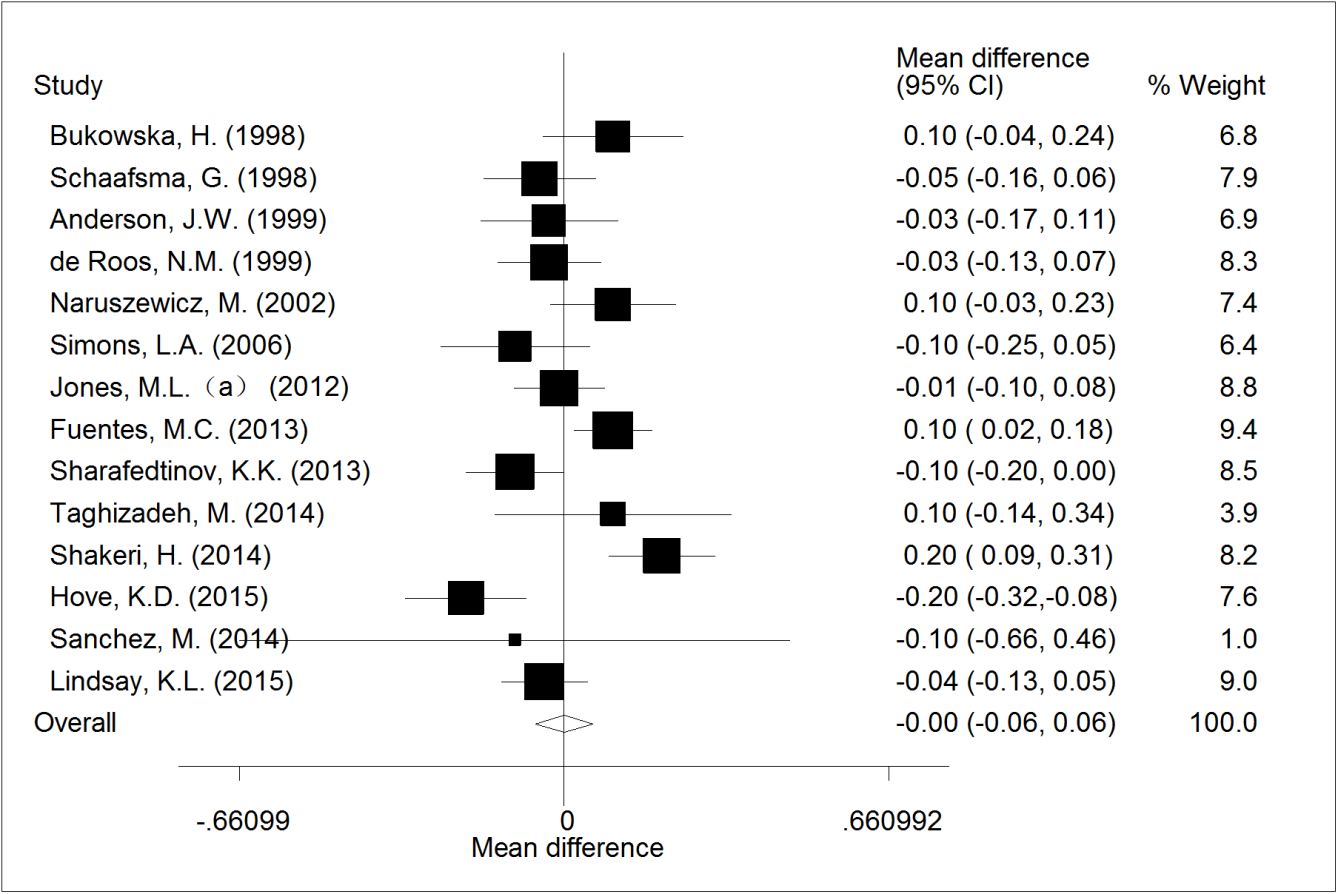
The effect of consumption of probiotic *Lactobacillus* on HDL.

Fourteen RCTs reported the effect of probiotic *Lactobacillus* onhigh density lipoprotein cholesterol (HDL-C) levels. A pooled effect was found to be nonsignificant (WMD = -0.00; CI,-0.06 to 0.06; p = 0.99; I^2^ = 68.7%; p = 0.008 for heterogeneity, Fig 5).

### Evaluation of subgroup analysis

A random-effects meta-analysis of 4 RCTs showed a significant decrease TC and LDL-C levels after the consumption of *L*. *plantarum*. (WMD = -0.37; CI, -0.60 to -0.13; p = 0.002; WMD = -0.29; CI, -0.43 to -0.16; p < 0.001; respectively, Table 2). The included studies showed highly significant homogeneity (I^2^ = 41.2%, p = 0.022; I^2^ = 0.0%, p <0.001, respectively).

**Table 2:** Evaluation of subgroup analysis.

Subgroup Analysis	Weight Mean Difference (95% Confidence Interval)	No. of study	p value
TG			
L. plantarum	0.05(-0.16, 0.26)	4	0.636
L. acidophilus	0.05(-0.09, 0.19)	3	0.504
L. fermentum	-0.10(-0.40, 0.20)	1	0.508
L. reuteri	-0.15 (-0.46,0.16)	1	0.346
L. sporogenes	-0.58(-0.97, -0.19)	2	0.003
L. salivarius	-0.04 (-0.26, 0.18)	1	0.717
L. rhamnosus	0.10(-0.74, 0.94)	1	0.816
All trials	-0.02(-0.10, 0.07)	13	0.481
TC			
L. plantarum	-0.37(-0.60, -0.13)	4	0.002
L. acidophilus	-0.15(-0.41, 0.12)	3	0.275
L. fermentum	-0.10(-0.49, 0.29)	1	0.613
L. reuteri	-0.58(-0.81, -0.35)	1	<0.001
L. sporogenes	-0.30(-0.74, 0.15)	2	0.189
L. helveticus	0.00(-0.41, 0.41)	1	1.000
L. salivarius	-0.23(-0.50, 0.04)	1	0.091
L. rhamnosus	-0.10(-1.47, 1.27)	1	0.886
All trials	-0.26(-0.40, -0.12)	14	<0.001
LDL-C			
L. plantarum	-0.29 (-0.43, -0.16)	4	<0.001
L. acidophilus	-0.14(-0.39, 0.11)	3	0.275
L. fermentum	0.00(-0.39, 0.39)	1	1.000
L. reuteri	-0.52(-0.65, -0.40)	2	<0.001
L. sporogenes	-0.13(-0.56, 0.31)	2	0.567
L. helveticus	0.20(-0.11, 0.51)	1	0.204
L. salivarius	-0.23(-0.49, 0.03)	1	0.082
L. rhamnosus	-0.10(-1.19, 0.99)	1	0.857
All trials	-0.23(-0.36, -0.10)	15	<0.001
HDL-C			
L. plantarum	0.05(-0.06, 0.15)	4	0.385
L. acidophilus	-0.04(-0.11, 0.03)	3	0.285
L. fermentum	-0.10(-0.25, 0.05)	1	0.198
L. reuteri	-0.01(-0.10, 0.08)	1	0.833
L. sporogenes	0.18(0.08, 0.28)	2	<0.001
L. helveticus	-0.20(-0.32, -0.08)	1	0.001
L. salivarius	-0.04(-0.13, 0.05)	1	0.375
L. rhamnosus	-0.10(-0.66,0.46	1	0.727
All trials	-0.00(-0.06, 0.06)	14	0.990

A pooled analysis of 2 RCTs showed a significant reduction in LDL levels receiving *L*. *reuteri* compared with those receiving placebo (WMD = -0.52; CI, -0.65 to -0.40; p <0.001; Table 2). The included studies showed highly significant homogeneity (I^2^ = 0.0%, p = 0.880).

A pooled analysis of 2 RCTs demonstrated a significant decrease in TG levels and increase in HDL-C levels after the use of the synbiotic food containing *L*. *sporogenes* and inulin compared with control groups (WMD = -0.58; CI, -0.97 to -0.19; p = 0.003; WMD = 0.18; CI, 0.08 to 0.28; p < 0.001; respectively, Table 2). The included studies showed highly significant homogeneity (I^2^ = 22.0%, p = 0.258; I^2^ = 0.0%, p = 0.460, respectively).

Besides, subgroup analysis with the givensubjects of non-pregnancy, non-diabetes/obesityordiabetes/obesitywas performed in supplemental material (S2 Table). Most of the results remained similar to the main outcomes, except LDL-C in the diabetes/obesity subjects became nonsignificant.

### Publication bias diagnostics

**Fig 6:**
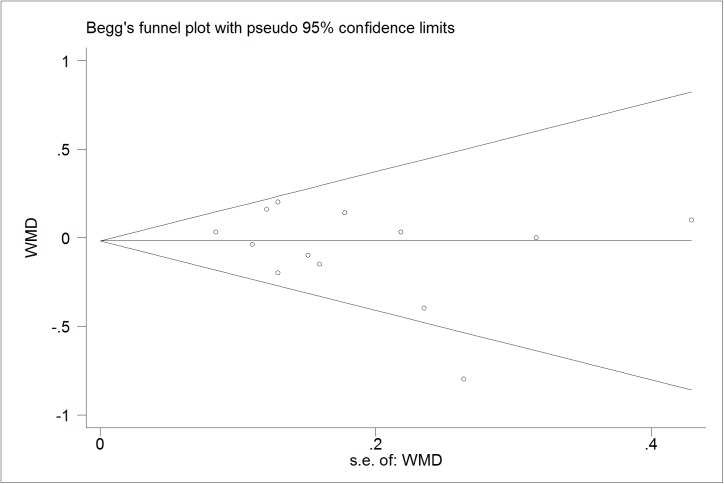
Begg’s funnel plot for publication bias in trials on the effect of probiotics *Lactobacillus* on TG.

**Fig 7:**
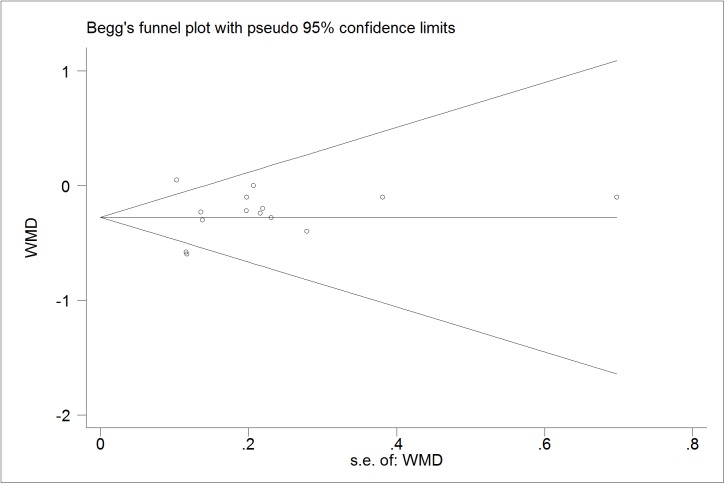
Begg’s funnel plot for publication bias in trials on the effect of probiotics *Lactobacillus* on TC.

**Fig 8:**
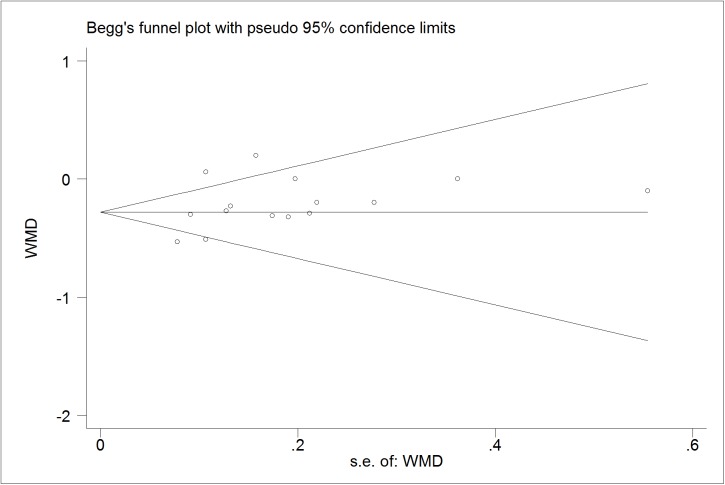
Begg’s funnel plot for publication bias in trials on the effect of probiotics *Lactobacillus* on LDL.

**Fig 9:**
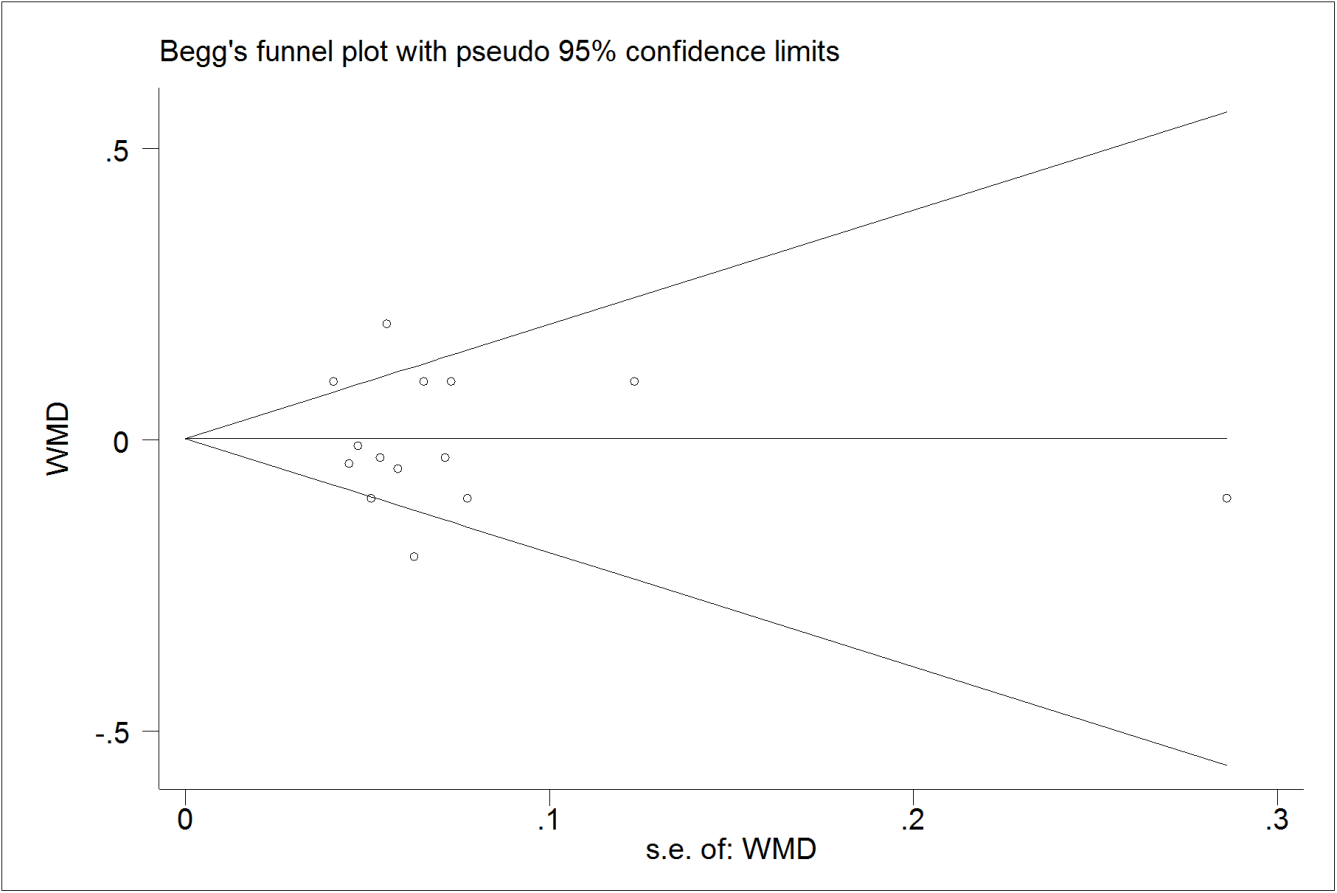
Begg’s funnel plot for publication bias in trials on the effect of probiotics *Lactobacillus* on HDL.

We further identify the potential publication biases of literatures by Egger’s test and Begg’s funnel plot. In all trials, the shapes of funnel plot indicated no obvious asymmetry (Figs 6–9). And Egger’s test provided statistical evidence for the funnel plot symmetry. No significant publication bias was found in the trials (p = 0.218 for TG; p = 0.599 for TC; p = 0.141 for LDL-C; p = 0.785 for HDL-C).

## Discussion

The effects of probiotics consumption on lipid control have attracted increasing interest recently [22, 23]. A meta-analysis of 13 randomized controlled trials reported that probiotics consumption was able to decrease total-cholesterol and LDL-cholesterol levels effectively [24].Previous studies show that the gut microbiome may play an important role in the variation in serum lipids, supporting the potential of therapies altering the gut microbiome to control triglycerides and high-density lipoproteins[25, 26]. While, there are many different probiotic strains on the market. It is of practical interest to know the effect of specific strains rather than probiotics as ‘a whole group’. So, we conducted this meta-analysis to explore the effects of probiotic *Lactobacillus* consumption on lipid profile.

This study demonstrated that consumption of *Lactobacillus* has beneficial effects on serum TC and LDL-C levels, while no obvious changes in serum HDL-C and TG levels. As we know, this is the first meta-analysis assessing the effect of probiotics *Lactobacillus* on lipid profile. The mechanisms of reducing cholesterol levels may be as following: reduction of the enterohepatic circulation of bile salts; assimilation of cholesterol in the gastrointestinal tract; production of propionic acid which could decrease blood lipids; conversion of cholesterol into coprostanol in the gut[27–29]. Furthermore, it appears that activation of FXRα, nuclear receptor of bile acids, can also improve triglyceride control in animal models[30]. Besides, activation of the bile acid cell membrane receptor TGR5 may also activate thyroid hormone in brown adipose tissue and muscle, which increases energy expenditure and therefore improves serum lipids[31, 32].

The present meta-analysis found that consumption of *L*. *reuteri* and *L*. *plantarum* could decrease the TC and LDL-C levels effectively. This is in accordance with the findings by Singh TP et al.[33]who reported that the values for TC and LDL-C of pigs were reduced significantly in group fed with *L*. *reuteri* LR6, and also in line with findings by Salaj R et al. [34]who showed that *Lactobacillus plantarum* LS/07 reduced TC and LDL in rats fed with a high fat diet. Few studies have explored the mechanisms of decreasing TC and LDL-C levels by *L*. *reuteri* and *L*. *plantarum*. Yamaoka-Tojo M et al.[35] provided possible mechanism that bile acid-binding resins decrease the concentration of bile acids returned to the liver via enterohepatic cycling and therefore stimulate the conversion of cholesterol to bile acids.

Moreover, in the present study, consumption of the synbiotic food, containing *L*. *sporogenes* and inulin, significantly decreased serum TG and increased serum HDL-C levels, while had no impact on serum TC and LDL-C compared with control. Similar findings have also been shown in hypercholesterolemic pigs fed with a synbiotic food containing *L*. *acidophilus*, fructooligosaccharide, inulin and mannitol for 8 weeks[36]. The mechanisms of decreasing TG and increasing HDL-C levels by synbiotics remained unclear. One of the possible mechanisms was inhibition of synthesis of fatty acids in the liver by producing short-chain fatty acid (SCFA)[37]. Next, inulin played a determinant role in decreasing expression of lipogenic enzymes[38]. Moreover, inulin and probiotics may have a synergistic effect when administered as a synbiotic[39]. Besides, we also found that consumption of *L*. *helveticus* significantly decreased serum HDL-C levels, reminding us that not all *Lactobacillus* are beneficial to health.

There are some limitations to this meta-analysis. Primarily, the bias of the included studies exists in some aspects such as the dietary restrictions and exercise. And crossover studies may bring additional biases. Besides, some studies had a small number of participants and a quite short duration of *Lactobacillus* consumption. Last, the insufficient databases were searched, and it was absence of papers in other languages and pre-published protocol. Further researches with larger sample and longer duration are required to verify the effect of probiotic *Lactobacillus* on lipid profile.

## Conclusion

This meta-analysis showed that consumption of probiotic *Lactobacillus*, especially *L*. *reuteri* and *L*. *plantarm*, could reduce TC and LDL-C significantly. The study also suggested significantly beneficial effects on TG and HDL-C by consuming synbiotic food, containing *L*. *sporogenes* and inulin. The effect of probiotic *Lactobacillus* on serum lipids, as well as their mechanisms involved, required further investigation. This study provides useful information on the effects of probiotic *Lactobacillus* on serum lipids, which could make a contribution to the application of probiotic *Lactobacillus*, especially *L*. *reuteri*, *L*. *plantarm* and synbiotics, as novel therapies in lipids control.

## Supporting information

S1 Table Risk of bias assessment of all included articles. + low risk of bias (plausible bias unlikely to seriously alter the results),—high risk of bias (plausible bias that seriously weakens confidence in the results),? unclear risk of bias (plausible bias that raises some doubt about the results).
(DOCX)

S2 Table Subgroup Analysis of non-pregnancy, non-diabetes/obesity and diabetes/obesity. (DOCX)

S3 Table The full-text excluded articles and the reasons for exclusion. (DOCX)

S1 File PRISMA 2009 flow diagram. (DOC)
