# Supplementary material for: Effect of probiotic Lactobacillus on lipid profile: A systematic review and meta-analysis of randomized, controlled trials
Source: PLoS One. 2017 Jun 8;12(6):e0178868. doi: 10.1371/journal.pone.0178868 (PMC5464580; doi:10.1371/journal.pone.0178868)
Supplement: S1 Table — + low risk of bias (plausible bias unlikely to seriously alter the results),—high risk of bias (plausible bias that seriously weakens confidence in the results),? unclear risk of bias (plausible bias that raises some doubt about the results). (DOCX) [file pone.0178868.s001.docx]

| References | Cochrane Collaboration tool | | | | | | |
| --- | --- | --- | --- | --- | --- | --- | --- |
|  | Randomization | Concealed allocation | Blinding of participants and personnel | Blinding of outcome assessment | Incomplete outcome data | Selective reporting | Other bias |
| Bukowska, H.  (1998) | ? | ? | + | ? | + | ? | + |
| Schaafsma, G.  (1998) | ? | ? | + | ? | + | ? | + |
| Anderson, J.W.  (1999) | ? | ? | + | ? | + | ? | + |
| de Roos, N.M.  (1999) | ? | ? | + | ? | + | ? | + |
| Naruszewicz, M.  (2002) | ? | ? | + | ? | + | ? | + |
| Simons, L.A.  (2006) | ? | ? | + | - | + | ? | + |
| Jones, M.L.(a)  (2012) | ? | ? | + | ? | + | ? | + |
| Jones, M.L.(b)  (2012) | ? | ? | + | ? | + | ? | + |
| Fuentes, M.C.  (2013) | + | ? | + | - | - | ? | - |
| Sharafedtinov, K.K.  (2013) | ? | ? | + | - | + | ? | + |
| Taghizadeh, M.  (2014) | ? | ? | + | - | + | ? | + |
| Shakeri, H.  (2014) | ? | ? | + | - | + | ? | + |
| Hove, K.D.  (2015) | + | ? | + | ? | + | ? | + |
| Lindsay, K.L.  (2015) | ? | ? | + | ? | + | ? | + |
| Sanchez, M.  (2014) | ? | ? | + | ? | + | ? | + |

S1 Table. Risk of bias assessment of all included articles.

Source of bias: selection bias (randomization and concealed allocation), performance bias (blinding of participants and personnel), detection bias (blinding of outcome assessment), attrition bias (incomplete outcome data: >20 %), reporting bias (selective reporting) and other source of bias.

+ low risk of bias (plausible bias unlikely to seriously alter the results), - high risk of bias (plausible bias that seriously weakens confidence in the results), ? unclear risk of bias (plausible bias that raises some doubt about the results).
