# Supplementary material for: Effect of probiotic Lactobacillus on lipid profile: A systematic review and meta-analysis of randomized, controlled trials
Source: PLoS One. 2017 Jun 8;12(6):e0178868. doi: 10.1371/journal.pone.0178868 (PMC5464580; doi:10.1371/journal.pone.0178868)
Supplement: S2 Table — (DOCX) [file pone.0178868.s002.docx]

| **Subgroup Analysis** | **Weight Mean Difference**  **(95% Confidence Interval)** | **No. of study** | **p value** |
| --- | --- | --- | --- |
| TG |  |  |  |
| non-pregnancy  non-diabetes/obesity  diabetes/obesity | -0.00(-0.10, 0.09)  -0.00(-0.12, 0.11)  -0.20(-0.60, 0.20) | 11  9  4 | 0.924  0.952  0.319 |
| TC |  |  |  |
| non-pregnancy  non-diabetes/obesity  diabetes/obesity | -0.27(-0.43, -0.11)  -0.28(-0.47, -0.09)  -0.20(-0.38, -0.02) | 12  9  5 | 0.001  0.004  0.032 |
| LDL-C |  |  |  |
| non-pregnancy  non-diabetes/obesity  diabetes/obesity | -0.24(-0.38, -0.09)  -0.28 (-0.42, -0.13)  -0.12(-0.33, 0.10) | 13  10  5 | 0.001  <0.001  0.286 |
| HDL-C |  |  |  |
| non-pregnancy  non-diabetes/obesity  diabetes/obesity | 0.00(-0.07, 0.07)  0.02( -0.03, 0.07)  -0.02(-0.15,0.12) | 12  9  5 | 0.973  0.474  0.831 |

S2 Table. Subgroup Analysis of non-pregnancy, non-diabetes/obesity and diabetes/obesity.
