# Supplementary material for: Effect of probiotic Lactobacillus on lipid profile: A systematic review and meta-analysis of randomized, controlled trials
Source: PLoS One. 2017 Jun 8;12(6):e0178868. doi: 10.1371/journal.pone.0178868 (PMC5464580; doi:10.1371/journal.pone.0178868)
Supplement: S3 Table — (DOCX) [file pone.0178868.s003.docx]

| PMID | reasons for exclusion |
| --- | --- |
| 26117402 | Incomplete information on outcomes |
| 25331262 | Incomplete information on outcomes |
| 22019281 | Incomplete information on outcomes |
| 18506924 | Incomplete information on outcomes |
| 15841092 | Incomplete information on outcomes |

S3 Table. The full-text excluded articles and the reasons for exclusion.
