# Supplementary material for: Effect of probiotic Lactobacillus on lipid profile: A systematic review and meta-analysis of randomized, controlled trials
Source: PLoS One. 2017 Jun 8;12(6):e0178868. doi: 10.1371/journal.pone.0178868 (PMC5464580; doi:10.1371/journal.pone.0178868)
Supplement: S1 File — (DOC) [file pone.0178868.s004.doc]

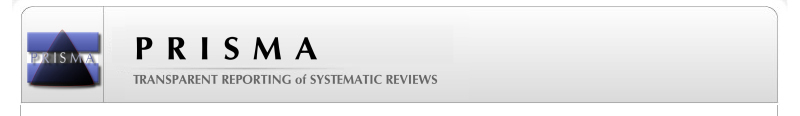
**PRISMA 2009 Flow Diagram**

**Screening**

**Included**

**Eligibility**

**Identification**

Records identified through database searching
(n = 85 )

Additional records identified through other sources
(n = 2 )

Records after duplicates removed
(n = 87 )

Records screened
(n = 37 )

Records excluded
(n = 17 ):Review; Not RCT;Not adults; Animal trials;Not English

Full-text articles assessed for eligibility
(n = 20 )

Full-text articles excluded, with reasons
(n = 5 ): Incomplete information on outcomes

Studies included in qualitative synthesis
(n = 15 )

Studies included in quantitative synthesis (meta-analysis)
(n = 15 )
